# Rivaroxaban versus enoxaparin plus clopidogrel therapy for hypertrophic cardiomyopathy-associated thromboembolism in cats

**DOI:** 10.14202/vetworld.2024.796-803

**Published:** 2024-04-10

**Authors:** Kotchapol Jaturanratsamee, Palin Jiwaganont, Chattida Panprom, Soontaree Petchdee

**Affiliations:** 1 Bio-Veterinary Science Program, Graduate School, Faculty of Veterinary Medicine, Kasetsart University, Kamphaeng Saen, Nakorn Pathom, Thailand; 2 Veterinary Clinical Studies Program, Graduate School, Faculty of Veterinary Medicine, Kasetsart University, Kamphaeng Saen, Nakorn Pathom, Thailand; 3 Department of Livestock Development, Supphaya District Livestock Office, Supphaya, Chai Nat, Thailand; 4 Department of Large Animal and Wildlife Clinical Sciences, Faculty of Veterinary Medicine, Kasetsart, University, Kamphaeng Saen, Nakorn Pathom, Thailand

**Keywords:** cardiomyopathy, D-dimer, echocardiography, prothrombin time

## Abstract

**Background and Aim:**

Cardiogenic embolism (CE) is a common complication of feline hypertrophic cardiomyopathy (HCM), leading to severe clinical symptoms. This study compared the effects of rivaroxaban and enoxaparin combined with clopidogrel on cats.

**Materials and Methods:**

This was a single-center, prospective, randomized controlled trial. In this study, rivaroxaban or enoxaparin plus clopidogrel was prescribed to 23 cats for at least one of the following events: Abnormal movement of the anterior mitral leaflet during systole, enlargement of the left atrium, spontaneous echocardiographic contrast, or presence of arterial thromboembolism. Oral rivaroxaban (2.5 mg, q24 h) was prescribed to six cats. Subcutaneous injections of enoxaparin (1 mg/kg, q24 h) plus oral clopidogrel (3 mg/kg, PO q24 h) for 60 days were administered to 17 cats. Renal insufficiency and bleeding complications were observed. Plasma concentrations of D-dimer, prothrombin time (PT), partial thromboplastin time, and international normalized ratio (INR) were evaluated. We analyzed the relationship between echocardiography parameters and the effects of coagulation. Blood samples were collected from all cats at baseline and at 1 and 2 months post-treatment.

**Results:**

Rivaroxaban alone and in combination with enoxaparin and clopidogrel significantly affected PT and INR. In cats treated with 2.5 mg/kg rivaroxaban for 60 days, no bleeding or recurrence of thrombus formation was observed. These data support the use of rivaroxaban for the treatment of HCM-associated thromboembolism in cats.

**Conclusion:**

Treatment of HCM-associated thromboembolism with rivaroxaban alone demonstrated clinical effectiveness with no clinical complications in cats.

## Introduction

Feline hypertrophic cardiomyopathy (HCM) usually presents with left ventricular stiffness, abnormal movement of the anterior mitral leaflet during systoles, diastolic relaxation disorder, enlargement of the left atrium (LA), and spontaneous echocardiographic contrast [1, 2]. Cardiomyopathy associated with arterial thromboembolism is a significant cause of thrombogenesis, which can partially or entirely block blood vessels [3]. The thrombus usually restricts blood flow to the lower extremities, provoking and causing tissue ischemia and paresis. In addition, blood clots can also be present in the main blood vessels of the kidneys, brain, and lungs and can cause serious problems or sudden death [4]. Clinical manifestations of pulmonary thromboembolism are often non-specific. Common symptoms include dyspnea, tachycardia, hypoxia, acute heart failure, and sudden death. Diagnosis is based on clinical findings, echocardiography imaging, and laboratory tests [5]. The D-dimer test is clinically used in human medicine to diagnose pulmonary embolism. Emergency treatment can be performed by administering thrombolytic drugs [6]. Previous studies have reported that cardiomyopathy-associated with thrombogenesis has a high mortality rate, with a 37% survival rate for cats with deep vein thrombosis. It is essential to prevent the recurrence of arterial thromboembolism. In addition, monitoring antithrombin therapy is a valuable tool for guiding drug administration and effective treatment regulation for each patient. Treatment monitoring enables the determination of the most appropriate therapy to achieve a clinical response in the absence of titration. Clopidogrel is widely used to prevent the onset or recurrence of thromboembolism in cats with HCM [7]. A previous study reported that clopidogrel at a total dose of 18.75 mg/cat significantly decreased platelet aggregation [8-10]. Low-molecular-weight heparin (LMWH) has replaced the use of unfractionated heparin in human and feline medicine because it can be injected subcutaneously, safely, and effectively for the initial treatment of venous thromboembolism [11, 12] because it is more convenient for clinical use.

However, the administration of low-molecular-weight antiplatelet agents in combination with clopidogrel and appropriate dose adjustment in cats with symptomatic cardiomyopathy has not yet been investigated. At present, available LMWH products in Thailand include enoxaparin, dalteparin, and bemiparin. Various LMWHs have different properties, especially chemical characteristics. The relative efficacy and safety of individual LMWH remain to be determined because few clinical trials have been performed [13–15]. Rivaroxaban is an oral anticoagulant that helps prevent and treat blood clots and is another option for human patients [16]. However, there is a need for more information on the use of this drug in cats. Antiplatelet therapy, such as rivaroxaban, has been reported to reduce the incidence of sudden cardiac death and myocardial infarction in humans [17, 18]. Dual therapy with clopidogrel and rivaroxaban has been shown to be effective in cat thromboprophylaxis [19]. The international normalized ratio (INR) test determines the clotting time. Detection of INR helps to indicate clotting time in patients receiving anticoagulants or blood-thinning agents such as enoxaparin [20].

This study aimed to evaluate and monitor the therapeutic response of rivaroxaban alone or clopidogrel in combination with an LMWH agent in cats with HCM. We hypothesized that rivaroxaban or LMWH plus clopidogrel would decrease recurrent thrombus formation in cats with HCM. The results of this study may help evaluate and monitor the treatment of cardiomyopathy-associated with arterial thromboembolism in cats.

## Materials and Methods

### Ethical approval

The study protocol was approved by the Ethics Committee of Kasetsart University (ACKU-62-VET-059) and written informed consent was obtained from the owners.

### Study period and location

We conducted a retrospective, single-center, randomized controlled trial from December 2022 to September 2024. Data were collected by convenience sampling. Twenty-three cats were recruited from the Animal Teaching Hospital Kamphaeng Saen, Faculty of Veterinary Medicine, Kasetsart University, with the owner’s complete informed consent.

### Animals

Cats at least 1 year old were recruited for inclusion in this study. Cats with structural heart abnormalities, hyperthyroidism (total T4 level >3.8 μg/dL), high blood pressure (systolic blood pressure >160 mmHg), pregnancy or breast-feeding periods, and cats with current chronic diseases, such as chronic kidney disease (CKD), diabetes, or epilepsy, were excluded from this study.

All cats underwent a physical examination including sex, breed, body weight, age, and medical history. The clinical records of each cat were obtained by transthoracic echocardiography and blood profile examination. Blood samples were collected from the cephalic, or medial saphenous veins.

In addition, 2 mL of blood was collected in a tube containing sodium citrate for coagulation marker evaluation. D-dimer levels and prothrombin time (PT) were immediately examined in the collected blood samples. Cats with significant hematological abnormalities, such as anemia, thrombocytopenia, leukocytosis, CKD, or previous anticoagulant therapy, were excluded. In cats with risk factors or signs indicating deep vein thrombosis, such as lower limb edema, ultrasonography of the inferior limb veins was performed.

### Echocardiography

**Figure-1:**
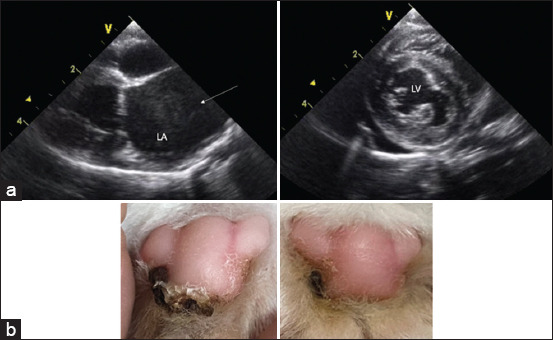
Echocardiographic assessment of left ventricular wall in hypertrophic cardiomyopathy cat. (a) Right parasternal long view of the left atrium and short-axis view of the left ventricle; The spontaneous echocardiographic contrast in the left atrium of a cat (arrow). (b) the cat’s paw before and after treatment with 1 mg/kg of enoxaparin and clopidogrel at 3 mg/kg for 60 days.

Cat presented with clinical signs such as acute paraparesis of the hind legs, idiopathic lesions on the pad (Figure-1b), and congestive heart failure were included in this study. Transthoracic echocardiography was performed using an ultrasound system (GE, Boston, MA, USA). We evaluated M-mode, 2D, pulse-wave, and continuous-wave Doppler measurements. HCM was assessed based on standard echocardiographic views described by Payne *et al*. [21]. Echocardiography revealed such as an interventricular septum or left ventricular free wall thickening and left atrial enlargement, spontaneous echocardiographic contrast in the LA, and echocardiography images are shown in Figures-1a.

### Adverse events and endpoints

Adverse reactions include bleeding and other serious adverse reactions, such as death and renal failure. The study’s endpoints were bleeding events, renal insufficiency, or sudden death.

### Statistical analysis

Data were presented as mean ± standard error of the mean. The data sets were analyzed using one-way analysis of variance or Student’s t-test to compare continuous variables between the two groups, and p < 0.05 was considered statistically significant. Pearson’s correlation test was used to assess correlations among variables. GraphPad Prism software (version 9.0, USA) was used for statistical analysis.

## Results

### Baseline characteristics of study animals

A total of 23 cats (aged 3.39 ± 0.59 years and weighing 4.27 ± 0.27 kg) were included in the study. Sudden death occurred in seven cats treated with enoxaparin plus clopidogrel and one cat with acute kidney injury (AKI).

### Echocardiographic parameters of the study animals

Table-1 shows the available echocardiographic parameters for all animals. Echocardiographic parameters such as LA, aorta (AO), LA/AO ratio, diastolic interventricular septum thickness (IVSd), left ventricular end-systolic diameter, and percentage of fractional shortening (FS) were not significantly different between the groups. Parameters IVSd, left ventricular wall diastolic thickness, systolic interventricular septum thickness, and left ventricular wall systolic thickness decreased following treatment. In addition, pulmonary valve maximum blood velocity and isovolumic relaxation time (IVRT) were reduced compared with baseline values.

**Table-1:** Echocardiographic parameters.

Parameters			
Numbers of cats	Baseline	1 month	2 months
Rivaroxaban	n = 6	n = 6	n = 6
Enoxaparin and clopidogrel	n = 17	n = 12	n = 9
IVSd (cm)			
Rivaroxaban	0.65 ± 0.06	0.61 ± 0.05	0.64 ± 0.06
Enoxaparin and clopidogrel	0.59 ± 0.02	0.54 ± 0.03	0.56 ± 0.03
LVIDd (cm)			
Rivaroxaban	1.37 ± 0.11	1.44 ± 0.13	1.41 ± 0.18
Enoxaparin and clopidogrel	1.37 ± 0.08	1.29 ± 0.08	1.36 ± 0.11
LVPWd (cm)			
Rivaroxaban	0.66 ± 0.06	0.59 ± 0.06	0.64 ± 0.06
Enoxaparin and clopidogrel	0.57 ± 0.02	0.55 ± 0.02	0.56 ± 0.04
IVSs (cm)			
Rivaroxaban	0.74 ± 0.07	0.66 ± 0.06	0.68 ± 0.07
Enoxaparin and clopidogrel	0.70 ± 0.03	0.65 ± 0.04	0.66 ± 0.04
LVIDs (cm)			
Rivaroxaban	0.77 ± 0.09	0.92 ± 0.11	0.80 ± 0.09
Enoxaparin and clopidogrel	0.73 ± 0.07	0.76 ± 0.06	0.86 ± 0.06
LVPWs (cm)			
Rivaroxaban	0.74 ± 0.06	0.64 ± 0.03	0.70 ± 0.07
Enoxaparin and clopidogrel	0.65 ± 0.02	0.65 ± 0.06	0.65 ± 0.03
FS (%)			
Rivaroxaban	44.09 ± 3.598	37.31 ± 2.33	40.50 ± 3.57
Enoxaparin and clopidogrel	46.90 ± 2.94	42.09 ± 2.56	41.88 ± 3.17
LA			
Rivaroxaban	1.16 ± 0.07	1.22 ± 0.07	1.19 ± 0.09
Enoxaparin and clopidogrel	1.35 ± 0.08	1.31 ± 0.07	1.24 ± 0.07
LA/AO			
Rivaroxaban	1.48 ± 0.08	1.51 ± 0.09	1.67 ± 0.11
Enoxaparin and clopidogrel	1.95 ± 0.12	1.85 ± 0.14	1.40 ± 0.07
PV PG (mmHg)			
Rivaroxaban	3.02 ± 0.32	2.53 ± 0.37	2.30 ± 0.21
Enoxaparin and clopidogrel	4.14 ± 0.59	3.36 ± 0.67	2.61 ± 0.37
MV E/A			
Rivaroxaban	0.90 ± 0.07	0.88 ± 0.07	0.84 ± 0.09
Enoxaparin and clopidogrel	1.13 ± 0.12	1.01 ± 0.07	0.94 ± 0.06
IVRT			
Rivaroxaban	0.06 ± 0.001	0.06 ± 0.001	0.06 ± 0.001
Enoxaparin and clopidogrel	0.07 ± 0.01	0.06 ± 0.001	0.05 ± 0.001

Data represented as mean ± standard error of the mean, IVSd=Diastolic interventricular septum thickness, IVSs=Systolic interventricular septum thickness, LVIDd=Left ventricular end-diastolic diameter, LVIDs=Left ventricular end-systolic diameter, LVPWd=Left ventricular wall diastolic thickness, LVPWs=Left ventricular wall systolic thickness, FS=Fractional shortening, LA=The left atrium, LA/AO=Left atrium and aorta ratio, MV E/A=Mitral valve leaflet E velocity per A velocity ratio, IVRT=Isovolumic relaxation time

### Correlation matrix of D-dimer levels, coagulation parameters, weight, and age in cats

**Figure-2:**
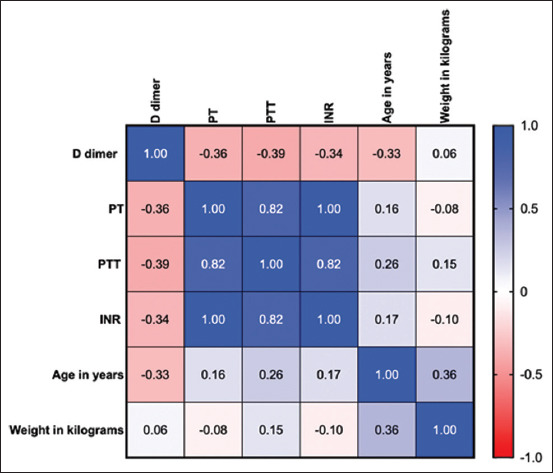
Heatmap of the correlation matrix generated by the Pearson r correlation coefficient for D-dimer, coagulation parameters, age, and weight. The scale is set from 0 (red) to 1 (blue). The Pearson correlation matrix shows the distribution and frequency of highly correlated individuals.

The coagulation factors PT, partial thromboplastin time (PTT), and INR were positively correlated with age and weight in cats with HCM (Figure-2). Interestingly, PT, PTT, and INR were positively correlated with age. However, PT, PTT, and INR were negatively correlated with the D-dimer.

### Correlation matrix of D-dimer levels, coagulation parameters, and echocardiography in cats

In this study, an antiplatelet agent (clopidogrel or rivaroxaban) for at least 2 months or extended therapy based on INR measurement provided effective thromboprophylaxis. After treatment, cats with thrombosis had lower plasma D-dimer levels and higher coagulation parameters (Table-2).

**Table-2:** The blood coagulation profiles in all cats.

Parameters (Mean ± standard error of the mean)	Baseline	1 month	2 months	Reference value
D-dimer (μg/mL)				
Rivaroxaban	0.31 ± 41.09	0.21 ± 20.62	0.16 ± 9.86	0–0.25
Enoxaparin and clopidogrel	0.38 ± 52.18	0.22 ± 34.51	0.16 ± 22.05	0–0.25
PT (s)				
Rivaroxaban	10.40 ± 0.32***	13.75 ± 1.28***	20.33 ± 1.28***	15.0–20.0
Enoxaparin and clopidogrel	9.68 ± 0.12***	10.68 ± 0.20***	11.69 ± 0.30***	15.0–20.0
PTT (s)				
Rivaroxaban	16.63 ± 0.52	17.35 ± 1.59	22.90 ± 1.07	15.0–21.0
Enoxaparin and clopidogrel	14.13 ± 0.45	15.31 ± 0.64	18.12 ± 0.59	15.0–21.0
INR				
Rivaroxaban	0.69 ± 0.02**	0.92 ± 0.08**	1.36 ± 0.09**	1.0–1.3
Enoxaparin and clopidogrel	0.64 ± 0.01**	0.72 ± 0.02**	0.80 ± 0.04**	1.0–1.3

PTT=Partial thromboplastin time, PT=Prothrombin time, INR=International normalized ratio,

** p < 0.01,

*** p < 0.001

**Figure-3:**
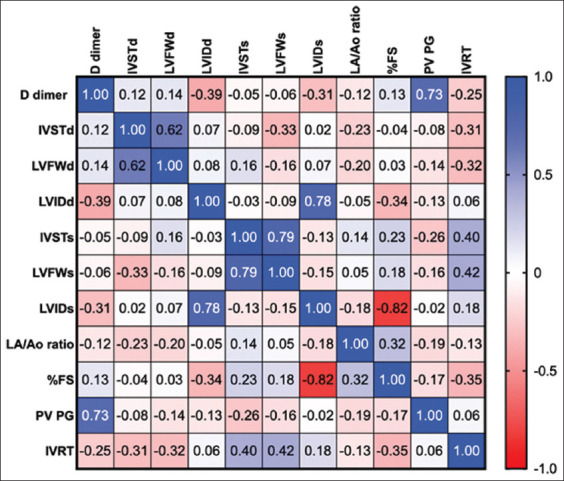
The correlation matrix of D-dimer and echocardiography among cats with hypertrophic cardiomyopathy. The scale is set from 0 (red) to 1 (blue), LA=Left atrium, AO=Aorta, IVSd=Interventricular septal at end-diastole, LVPWd=Left ventricular free proximal wall diameter at end-diastole, LVIDd=Left ventricular internal diameter at end-diastole, IVSs=Interventricular septal at end-systole, LVPWs=Left ventricular free proximal wall diameter at end-systole, LVIDs=Left ventricular inner diameter at end-systole, FS=Fractional shortening, PV Vmax=Pulmonary valve maximum blood velocity, AV Vmax=Aortic valve maximum blood velocity, FS=Fractional shortening, IVRT=Isovolumic relaxation time.

**Figure-4:**
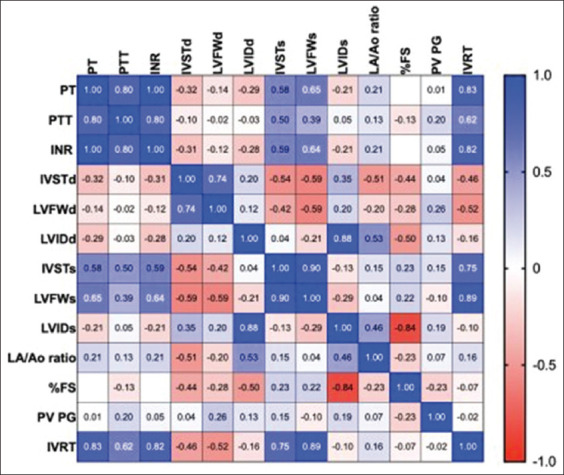
The correlation matrix of for coagulation (PT, PTT, and INR) and echocardiography among cats with hypertrophic cardiomyopathy. The scale is set from 0 (red) to 1 (blue), LA=Left atrium, AO=Aorta, IVSd=Interventricular septal at end-diastole, LVPWd=Left ventricular free proximal wall diameter at end-diastole, LVIDd=Left ventricular internal diameter at end-diastole, IVSs=Interventricular septal at end-systole, LVPWs=Left ventricular free proximal wall diameter at end-systole, LVIDs=Left ventricular inner diameter at end-systole, FS=Fractional shortening, PV Vmax=Pulmonary valve maximum blood velocity, AV Vmax=Aortic valve maximum blood velocity, FS=Fractional shortening, IVRT=Isovolumic relaxation time, PTT=Partial thromboplastin time, PT=Prothrombin time, INR=International normalized ratio.

A correlation between D-dimer and left ventricular wall thickness was observed. D-dimer is strongly correlated with the maximum blood velocity of the pulmonary valve, as shown in Figure 3. Correlation matrix between coagulation parameters and echocardiography response in cats with HCM. PT, PTT, and INR were strongly correlated with IVRT, interventricular septum, and left ventricular wall thickness during systole (Figure-4).

## Discussion

Myocardial wall stress abnormalities in HCM lead to mortality and scarring of the myocardium, eventually resulting in stiffening of the heart muscle, diastolic dysfunction, increased susceptibility to unstable electricity, and sudden death [22, 23]. Cardiogenic embolism is the most common complication in cats with cardiomyopathy and can lead to thromboembolism. Many factors, such as hypertension, diabetes, cancer, and cardiovascular disease, contribute to the occurrence of thromboembolism, including LA dilation. Recent studies have shown that fibrin plays a role in the growth of cardiomyocytes and cardiomyopathy and is stimulated in the right ventricle of rats during pulmonary thromboembolism [24, 25]. Not all of these factors can be avoided in cardiomyopathies.

Platelet activation plays a crucial role in thromboembolism. Therefore, antiplatelet therapy is crucial for treating cardiogenic thromboembolism (CE). However, platelet oxidase inhibitors, such as aspirin, may damage the gastrointestinal mucosa and cause gastrointestinal injury.

LMWH, such as enoxaparin, is used as a first-line anticoagulant. Because of its predictable pharmacokinetics, it is the gold standard for anticoagulant reduction and thrombosis prevention and has been widely used in clinical practice [26, 27]. Enoxaparin was used in the present study because of its action to prevent thromboembolic events, including therapeutic implications for the management of patients with myocardial disease. In addition, enoxaparin is available commercially. In addition, the Food and Drug Administration used *in vitro* and *ex vivo* to ensure that the enoxaparin product does not increase the risk of immunogenicity. However, previous studies have been conducted in healthy cats. Further studies are required to determine the optimal use in cats that effectively prevents thromboembolism and reduces the severity of thromboembolism in cats with cardiomyopathy [28, 29]. Treatment of cats with cardiomyopathy with LMWH agents may help prevent CE formation.

P2Y12 receptor antagonists, such as clopidogrel, also increase the risk of gastrointestinal bleeding. Along with aspirin, clopidogrel can make gastrointestinal injury more severe [28]. Previous studies have demonstrated the safety and efficacy of clopidogrel along with rivaroxaban in cats with thromboembolic disease [18]. In this study, one cat developed AKI during treatment with enoxaparin combined with clopidogrel. A previous study suggested that anticoagulation may cause glomerular overperfusion and kidney damage. The results of previous studies suggested that kidney function should be assessed while starting anticoagulant therapy and during follow-up treatment to determine renal risk [30].

Rivaroxaban is a direct factor Xa inhibitor that has advantages over warfarin in terms of efficacy and safety in diseases with a high risk of thromboembolism. Inhibition of factor Xa by low-dose rivaroxaban improves cardiovascular outcomes in human medicine rivaroxaban has advantages such as rapid onset of action, predictable efficacy, and no need for routine coagulation monitoring [31, 32]. In a previous study, rivaroxaban 15 mg once daily plus clopidogrel was shown to be a feasible treatment strategy for cats. The results of our study suggest that rivaroxaban 2.5 mg once daily for 60 days may provide a new approach for preventing CE in terms of efficacy and safety.

However, in cats with acute renal insufficiency, rivaroxaban should be initiated at 2.5 mg daily for 1 week. Dosing should be based on INR measurements, and renal function should be monitored. In cats treated with enoxaparin with bleeding complications, it is necessary to rapidly reverse anticoagulation with PT to adjust the dose of enoxaparin.

D-dimer measurements are increasingly used in veterinary practice for clinical reasons [33, 34]. Previous studies have compared plasma concentrations of the thrombin–antithrombin complex, D-dimer, and fibrin degradation products between healthy cats and cats with HCM [8]. However, further studies are needed to allow the clinical use of D-dimer in cats with HCM. This study found that a decrease in thrombus size is associated with D-dimer level. The results of this study are similar to those of a previous study where plasma D-dimer levels were higher in cats with HCM [8]. D-dimer levels were elevated in cats with HCM and CE, and cats with elevated D-dimer levels had lower FS and cardiac output.

Ultrasonography and D-dimer levels are recommended for confirmation in cats with suspected lower extremity thrombosis and cats with acute leg thrombosis who can walk.

The ages of the cats in our study were close to those reported in a previous study, which ranged between 3 and 6 years. Similar to the results of the previous study, the results of our study also showed a trend toward male predisposition to HCM (Table-3). In addition, elevated PT, PTT, and INR were found to be positively correlated with age and weight in our cat population (Figure-2).

**Table-3:** Characteristics of cats.

Parameters (Mean ± standard error of the mean)	Total (n = 23)	Rivaroxaban (n = 6)	Enoxaparin and clopidogrel (n = 17)
Age (years)	3.39 ± 0.59	2.83 ± 0.37	3.59 ± 0.79
Weight (kg)	4.27 ± 0.27	4.28 ± 0.48	4.26 ± 0.32
Male (n%)	65	66.67	64.71
Open mouth breathing (n%)	65	66.67	64.71
Paresis (n%)	34.78	50	29.41

Heart dimensions, such as left atrial enlargement, have been reported to be associated with survival rates in cats with HCM. However, our study found that an increased left ventricular wall thickness and an increase in IVRT in cats with HCM were associated with severe clinical signs of CE. Cats with cardiomyopathy and CE tended to have lower PT, PTT, and INR. Similar to previous studies, shortened PT significantly increased the incidence of thrombosis [35]. PT is used to evaluate endogenous and general coagulation pathways, and coagulation profile testing is necessary to evaluate coagulopathy in cats [36]. This study showed that PT and INR increased significantly after enoxaparin and clopidogrel administration. In addition, we found an association between shortened PT and thrombus size, suggesting that PT may be a helpful parameter to evaluate the clotting ability and the risk of venous thrombosis or clot-busting on paws associated with cardiomyopathy in cats.

## Limitation

A limitation of this study is that only a small number of cats were included in this research. In addition, the environment and nutrition in these cat populations cannot be controlled, which limits our study. This study is currently under recruitment. In December 2022, the first cat was included, and the recruitment will be completed in September 2024. At present, 44 cats have been recruited and 32 have completed 6 months of follow-up visits.

## Conclusion

This study suggested that rivaroxaban at 2.5 mg/kg or enoxaparin at 1 mg/kg and clopidogrel at 3 mg/kg for 60 days prevented thrombus formation in cats with arterial thromboembolism. Rivaroxaban 2.5 mg/kg once daily significantly decreased cardiac failure symptoms. The results of this study may provide a new approach for preventing thromboembolism associated with HCM in cats. In the future, the safety or efficacy of antithrombotic therapy should be further studied to evaluate indications for clinical application, including estimates of survival time for this antithrombotic therapy.

## Authors’ Contributions

SP: Identified the research topic and study area, performed the study, and drafted the manuscript. KJ, PJ, and CP: Analyzed and interpreted the data and revised the manuscript. All authors have performed the follow up. All authors have read, reviewed, and agreed to the published version of the manuscript. SP and CP: Performed and followed up the study.

## References

[1] Kittleson M.D, Côté E (2021). The feline cardiomyopathies:Hypertrophic cardiomyopathy. J. Feline Med. Surg.

[2] Sukumolanan P, Petchdee S (2022). Prevalence of cardiac myosin-binding protein C3 mutations in Maine Coon cats with hypertrophic cardiomyopathy. Vet World.

[3] D'Alessandro E, Winters J, van Nieuwenhoven F.A, Schotten U, Verheule S (2022). The complex relation between atrial cardiomyopathy and thrombogenesis. Cells.

[4] Brugada-Terradellas C, Hellemans A, Brugada P, Smets P (2021). Sudden cardiac death:A comparative review of humans, dogs and cats. Vet J.

[5] De Lima G.V, Ferreira F.D.S (2017). N-terminal-pro brain natriuretic peptides in dogs and cats:A technical and clinical review. Vet World.

[6] Mabrouk B, Anis C, Hassen D, Leila A, Daoud S, Hichem K, Mohamed S, Hatem K, Mounir B (2014). Pulmonary thromboembolism:Incidence, physiopathology, diagnosis and treatment. Tunis Med.

[7] Alonso Martínez J.L, Abínzano Guillén M.L, Solano Remírez M, Alvarez Frías M.T, Gutiérrez Dubois J, Munuera García L (2005). Low-molecular-weight heparin for the treatment of acute pulmonary thromboembolism. Comparison with unfractionated intravenous heparin. An. Med. Interna.

[8] Bédard C, Lanevschi-Pietersma A, Dunn M (2007). Evaluation of coagulation markers in the plasma of healthy cats and cats with asymptomatic hypertrophic cardiomyopathy. Vet. Clin. Pathol.

[9] Sharp C.R, deLaforcade A.M, Koenigshof A.M, Lynch A.M, Thomason J.M (2019). Consensus on the rational use of antithrombotics in veterinary critical care (CURATIVE):Domain 4-refining and monitoring antithrombotic therapies. J. Vet. Emerg. Crit. Care (San Antonio).

[10] Hamel-Jolette A, Dunn M, Bédard C (2009). Plateletworks:A screening assay for clopidogrel therapy monitoring in healthy cats. Can. J. Vet. Res.

[11] Ho K.K, Abrams-Ogg A.C, Wood R.D, O'Sullivan M.L, Kirby G.M, Blois S.L (2017). Assessment of platelet function in healthy cats in response to commonly prescribed antiplatelet drugs using three point-of-care platelet function tests. J. Feline Med. Surg.

[12] Blais M.C, Bianco D, Goggs R, Lynch A.M, Palmer L, Ralph A, Sharp C.R (2019). Consensus on the rational use of antithrombotics in veterinary critical care (Curative):Domain 3-defining antithrombotic protocols. J. Vet. Emerg. Crit. Care (San Antonio).

[13] Parvizi J, DeMik D.E, Dunbar M, Hozack W.J, Mont M.A, Lachiewicz P.F (2022). Low-molecular-weight heparin is superior to aspirin in the prevention of thromboembolic disease:Or is it?. J. Bone Joint Surg. Am.

[14] Koch A, Ziegler S, Breitschwerdt H, Victor N (2001). Low-molecular weight heparin and unfractionated heparin in thrombosis prophylaxis:Meta-analysis based on original patient data. Thromb. Res.

[15] Lim W, Dentali F, Eikelboom J.W, Crowther M.A (2006). Meta-analysis:Low-molecular-weight heparin and bleeding in patients with severe renal insufficiency. Ann. Intern. Med.

[16] Turpie A.G (2012). Advances in oral anticoagulation treatment:The safety and efficacy of rivaroxaban in the prevention and treatment of thromboembolism. Ther. Adv. Hematol.

[17] Xie S, Chen J, Xiong G, Li J, Wan J, Liu Y, Xu R, Zhang W (20021). The efficacy and safety of rivaroxaban in coronary artery disease patients with heart failure and sinus rhythm:A systematic review and meta-analysis. Eur. J. Clin. Pharmacol.

[18] Cho S.W, Franchi F, Angiolillo D.J (2019). Role of oral anticoagulant therapy for secondary prevention in patients with stable atherothrombotic disease manifestations. Ther. Adv. Hematol.

[19] Lo S.T, Walker A.L, Georges C.J, Li R.H, Stern J.A (2022). Dual therapy with clopidogrel and rivaroxaban in cats with thromboembolic disease. J. Feline Med. Surg.

[20] Shah Z, Mastoris I, Acharya P, Rali A.S, Mohammed M, Sami F, Ranka S, Wagner S, Zanotti G, Salerno C.T, Haglund N.A, Sauer A.J, Ravichandran A.K, Abicht T (2020). The use of enoxaparin as bridge to therapeutic INR after LVAD implantation. J. Cardiothorac. Surg.

[21] Payne J.R, Borgeat K, Connolly D.J, Boswood A, Dennis S, Wagner T, Menaut P, Maerz I, Evans D, Simons V.E, Brodbelt D.C, Fuentes L.V (2013). Prognostic indicators in cats with hypertrophic cardiomyopathy. J. Vet. Intern. Med.

[22] Payne J.R, Borgeat K, Brodbelt D.C, Connolly D.J, Luis Fuentes V (2015). Risk factors associated with sudden death vs. congestive heart failure or arterial thromboembolism in cats with hypertrophic cardiomyopathy. J. Vet. Cardiol.

[23] Saito T, Suzuki R, Yuchi Y, Fukuoka H, Satomi S, Teshima T, Matsumoto H (2023). Comparative study of myocardial function in cases of feline hypertrophic cardiomyopathy with and without dynamic left ventricular outflow-tract obstruction. Front. Vet. Sci.

[24] Fareed J, Jeske W, Fareed D, Clark M, Wahi R, Adiguzel C, Hoppensteadt D (2008). Are all low-molecular weight heparins equivalent in the management of venous thromboembolism?. Clin. Appl. Thromb. Hemost.

[25] Petersen M, Schmiedel N, Dierck F, Hille S, Remes A, Senger F, Schmidt I, Lüllmann-Rauch R, Müller O.J, Frank D, Rangrez A.Y, Frey N, Kuhn C (2023). Fibin regulates cardiomyocyte hypertrophy and causes protein-aggregate-associated cardiomyopathy *in vivo*. Front. Mol. Biosci.

[26] Yin X, Liu P, Liu B.Y, Liu Y.Y, Fan W.L, Zhao J.H (2018). Preventive effects of low-molecular weight heparin on formation of deep vein thrombosis by reducing D-dimer values in patients undergoing spinal surgery. Eur. Rev. Med. Pharmacol. Sci.

[27] Lee S, Raw A, Yu L, Lionberger R, Ya N, Verthelyi D, Rosenberg A, Kozlowski S, Webber K, Woodcock J (2013). Scientific considerations in the review and approval of generic enoxaparin in the United States. Nat. Biotechnol.

[28] Deitcher S.R (2000). Overview of enoxaparin in the treatment of deep vein thrombosis. Am. J. Manag. Care.

[29] Holzheimer R.G (2004). Low-molecular-weight heparin (LMWH) in the treatment of thrombosis. Eur. J. Med. Res.

[30] Zeni L, Manenti C, Fisogni S, Terlizzi V, Verzeletti F, Gaggiotti M, Cancarini G (2020). Acute kidney injury due to anticoagulant-related nephropathy A suggestion for therapy. Case Rep. Nephrol.

[31] Scott L.J (2020). Rivaroxaban:A review for secondary CV prevention in CAD and PAD. Drugs.

[32] Mega J.L, Braunwald E, Wiviott S.D, Bassand J.P, Bhatt D.L, Bode C (2012). Rivaroxaban in patients with a recent acute coronary syndrome. N. Engl. J. Med.

[33] Stokol T (2003). Plasma D-dimer for the diagnosis of thromboembolic disorders in dogs. Vet. Clin. North Am. Small Anim. Pract.

[34] Monreal L (2003). D-dimer as a new test for the diagnosis of DIC and thromboembolic disease. J. Vet. Intern Med.

[35] Song J, Drobatz K.J, Silverstein D.C (2016). Retrospective evaluation of shortened prothrombin time or activated partial thromboplastin time for the diagnosis of hypercoagulability in dogs:25 cases (2006–2011). J. Vet. Emerg. Crit. Care (San Antonio).

[36] Tonthong S, Rungpupradit J (2020). Coagulation testing:Comparison of portable (CoaguChek®XS) and automated coagulation analyzer in healthy cats. Vet. World.

